# Intracerebroventricular Administration of Interferon-Alpha Induced Depressive-Like Behaviors and Neurotransmitter Changes in Rhesus Monkeys

**DOI:** 10.3389/fnins.2020.585604

**Published:** 2020-11-19

**Authors:** Zhifei Li, Zhaoxia Li, Xiaoman Lv, Zhaofu Li, Lei Xiong, Xintian Hu, Dongdong Qin

**Affiliations:** ^1^Key Laboratory of Animal Models and Human Disease Mechanisms of the Chinese Academy of Sciences and Yunnan Province, Kunming Institute of Zoology, Chinese Academy of Sciences, Kunming, China; ^2^Yunnan University of Chinese Medicine, Kunming, China; ^3^National Resource Center for Non-Human Primates, Kunming Primate Research Center, National Research Facility for Phenotypic & Genetic Analysis of Model Animals (Primate Facility), Kunming Institute of Zoology, Chinese Academy of Sciences, Kunming, China; ^4^Kunming Primate Research Center, Kunming Institute of Zoology, Chinese Academy of Sciences, Kunming, China; ^5^Center for Excellence in Brain Science and Intelligence Technology, Chinese Academy of Sciences, Shanghai, China

**Keywords:** interferon-alpha, intracerebroventricular administration, rhesus monkeys, depressive-like behaviors, neurotransmitters

## Abstract

Interferon-alpha (IFN-α) is a cytokine widely used in the treatment of brain cancers and virus infections with side effects including causing depression. Monoamine neurotransmitter systems have been found playing important roles in peripheral IFN-α-induced depression, but how peripheral IFN-α accesses the central nervous system and contributes to the development of depression is poorly known. This study aimed to develop a non-human primate model using long-term intracerebroventricular (i.c.v.) administration of IFN-α (5 days/week for 6 weeks), to observe the induced depressive-like behaviors and to explore the contributions of monoamine neurotransmitter systems in the development of depression. In monkeys receiving i.c.v. IFN-α administration, anhedonia was observed as decreases of sucrose consumption, along with depressive-like symptoms including increased huddling behavior, decreases of spontaneous and reactive locomotion in home cage, as well as reduced exploration and increased motionless in the open field. Chronic central IFN-α infusion significantly increased the cerebrospinal fluid (CSF) concentrations of noradrenaline (NA), and 3,4-dihydroxyphenylacetic acid (DOPAC), but not 5-hydroxyindoleacetic acid (5-HIAA) and homovanillic acid (HVA). These CSF monoamine metabolites showed associations with some specific depression-related behaviors. In conclusion, central IFN-α administration induced anhedonia and depression-related behaviors comparable to the results with peripheral administration, and the development of depression was associated with the dysfunction of monoamine neurotransmitters.

## Introduction

Interferon-alpha (IFN-α) is a pleiotropic cytokine released in response to viral infection, which enhances the cellular immune response. IFN-α is usually used to treat certain cancers and infectious diseases because of its antiproliferative and antiviral effects. For example, intraventricular administration of IFN-α was used to treat central nervous system (CNS) diseases induced by viral infection such as subacute sclerosing panencephalitis (SSPE) (Yalaz et al., 1992; Kwak et al., 2019). However, 30–50% of the patients receiving IFN-α treatment suffer from major depressive disorder (MDD) (Hepgul et al., 2018; Su et al., 2019). Recruitment of cytokines including the IFN-α during CNS inflammation also contributes to the development of depression (Bodnar et al., 2018; Su et al., 2019).

How peripherally administrated IFN-α (e.g., intravenous and intramuscular injection) induces depression has been well studied in animal models including rodents (Hoyo-Becerra et al., 2015) and non-human primates (Felger et al., 2007). Rhesus monkeys receiving chronic (4 weeks) subcutaneous IFN-α administration showed increased anxiety-like and depression-like behaviors as well as diminished environmental exploration, accompanied by increased levels of plasma adrenocorticotropic hormone (ACTH) and cortisol, which tended to improve after the administration terminated. Monkeys that showed IFN-α-induced huddling behavior had lower cerebrospinal fluid (CSF) concentrations of homovanillic acid (HVA), a dopamine metabolite (Felger et al., 2007).

The study of mechanisms underlying IFN-α-induced depression has been largely focused on the monoamine neurotransmitter systems (Lotrich et al., 2009; Felger and Miller, 2012; Ping et al., 2012; Felger et al., 2013b). Monoamine neurotransmitters in the brain, especially serotonin (5-HT), play critical roles in the development of depression. Most antidepressants target at the functioning of 5-HT, such as selective serotonin reuptake inhibitors (SSRIs). Studies in both humans and laboratory animals have found that IFN-α administration can disrupt the functions of monoamine neurotransmitters by decreasing their synthesis and release (Anderson et al., 2013; Felger and Lotrich, 2013; Felger et al., 2013a), inhibiting their binding to the receptors (Ping et al., 2012; Felger et al., 2013b) and/or upregulating the reuptake from synapses (Tsao et al., 2008; Capuron et al., 2012).

It should be noted that IFN-α was peripherally administrated in most studies, and they are relatively large molecules (∼15–25 kD) that do not freely pass through the blood–brain barrier (BBB) (Collins et al., 1985; Smith et al., 1985), while some studies have reported that IFNs can enter the CNS from the periphery but with regional differences, and the spinal cord had greater permeability to IFNs than did the brain (Pan et al., 1997). Therefore, much attention should be paid to the pathways by which peripheral IFN-α signals reach the brain. Laboratory studies have shown that cytokines administered peripherally can access the brain through several routes including (1) passage through leaky regions in the BBB, (2) active transport via saturable transport molecules, (3) activation of endothelial cells (as well as other cells lining the cerebral vasculature), which then release inflammatory mediators within the brain parenchyma, and (4) binding to cytokine receptors associated with peripheral afferent nerve fibers (e.g., the vagus nerve) which in turn relay signals to relevant brain nuclei (Goehler et al., 2000; Quan and Banks, 2007; Dantzer et al., 2008). Nevertheless, it remains to be established whether peripheral IFN-α can access the brain and affect the brain functioning. Moreover, it has yet to be determined whether IFN-α-induced changes in behavior in humans are related to effects of central cytokines on the metabolism of behaviorally relevant neurotransmitter systems. Thus, it will be interesting to know: (1) whether direct administration of IFN-α into the brain can cause similar depression-like behaviors in rhesus monkeys as observed with peripheral administration; and (2) whether IFN-α can induce changes in monoamine neurotransmitters and contribute to the development of depression. In this study, IFN-α was directly delivered into the CNS via intracerebroventricular (i.c.v) administration. We analyzed the effects of central IFN-α on anhedonia, depression-related behaviors in both home cage and the open field, as well as the CSF concentrations of monoamine neurotransmitters and their metabolites.

## Materials and Methods

### Animals

All animal care and experimental protocols were approved by the National Animal Research Authority (China) and the Institutional Animal Care and Use Committee (IACUC) of Kunming Institute of Zoology, Chinese Academy of Sciences. Eight male rhesus monkeys (*Macaca mulatta*) ranging from 7 to 10 years old (8.00 ± 1.20, mean ± SD) were purchased from Kunming Primate Research Center of the Chinese Academy of Sciences. All monkeys were individually housed in a stainless-steel cage (80 × 80 × 80 cm) on the same side of the housing room with relatively constant temperature of 21 ± 2°C. Artificial illumination (800 lx) was provided from 07:00 to 19:00. Monkeys were supplied with commercial monkey biscuits and fruits, as well as *ad libitum* access to tap water, except for the days of sucrose preference test. Routine veterinary care was provided throughout the experiment by professional keepers and veterinarians. All possible efforts were made to minimize the suffering of animals.

### Surgical Procedures

After 1 week of habituation to the housing environment, all animals received the surgery of intraventricular intubation. The surgical procedures have been described in previous studies (Li et al., 2015; Zhai et al., 2018). Animals were anesthetized with intramuscular injections of atropine (20 mg/kg), ketamine (10 mg/kg), and sodium pentobarbital (20 mg/kg). Anesthesia was confirmed by pinching toes of the monkey, before fixing its head on a stereotaxic instrument (Model 1504 Heavy-Duty Research Stereotaxic for Monkeys, KOPF, United States). The skull over the parietal lobe was exposed by a longitudinal incision of the scalp, and tissues on the skull were removed. A small hole (about 2 mm in diameter) in the skull was drilled (anterioposterior: + 17 mm; mediolateral: −2 mm, with reference to the midpoint of the interaural line). A stainless-steel cannula (21 gauge, 40 mm, New England Small Tube Corporation, United States) was vertically inserted through the hole into the right lateral ventricle with depths ranging from 18 to 22 mm beneath the surface of the skull. Outflow or pulsation of the CSF at the orifice indicated that the cannula had reached the lateral ventricle. The cannula was then blocked with a removable stainless-steel plug, and protected with a truncated plastic centrifuge tube (15 ml) with the cap. Three titanium alloy screws were fixed on the skull surface to anchor the dental cement to the skull. Postoperative care was conducted by a veterinarian with daily observation and intramuscular injections of Penicillin (1,600 K Unit, Harbin Pharmaceutical Group Sixth Pharm Factory, Harbin, China) for 7 days, before any behavioral observation and i.c.v. administration.

### Intraventricular Administration of IFN-α

Postoperative monkeys were trained to sit in a customized monkey chair until they were habituated to the manipulations of intraventricular injection without anesthesia and experience of stress. All monkeys were then randomly separated into two administration groups with four animals in each group. Recombinant human IFN-α (1.4 × 10^5^ IU/kg bodyweight) or saline in equivalent volume was injected into the lateral ventricle gradually over a period of 15 min between 9:30 and 12:00 for 6 weeks (5 days/week). On the first and second administration day, the injected doses were, respectively, 1/4 and 1/2 of the normal dose in order to adapt monkeys to the drugs. A stainless-steel needle (42 mm) connected to a syringe with silicone capillary tube was inserted into the cannula for the injection. The injection was conducted with a microinjection pump (Smith WZ-50C6, Zhejiang Smith Medical Instrument Co Ltd, Hangzhou, China) when the animal was sitting awake in the monkey chair. The injection needle was retained in place for 5 min after the completion of injection.

### Sucrose Preference Tests

Sucrose preference tests were conducted in three 3-week phases, with 14 tests in each phase, namely, preadministration (Pre), the first half (Adm1) and second half (Adm2) of the administration period. Each phase lasted for 3 weeks, and the sucrose preference test was measured in a water-deprived state to maximize the motivation of monkeys. Briefly, a 1.5% sucrose solution and tap water was available in two adjacent bottles fixed to every home-cage from 8:30 to 9:30 every day, with no extra water available during the days of sucrose preference tests. Positions of these two liquids were switched every day to avoid the potential effects of position preference (Paul et al., 2000; Qin et al., 2015a). The consumed volume of each liquid was quantified and recorded. The sucrose preference was calculated as a percentage of consumed sucrose solution of the total amount of water drunk. Sucrose water and tap water intake were, respectively, calculated as ml consumed per kg body weight.

### Behaviors in Home Cage

Each monkey was videotaped for 30 min a day. Before the administration, the video recording lasted for 14 days. After completion of the injection, each monkey was again videotaped for another 14 days (30 min per day). The video recordings were evenly distributed in the light phase, with 3.5 h in the morning (8:00–8:30 a.m., 8:30–9:00 a.m., 9:00–9:30 a.m., 9:30–10:00 a.m., 10:00–10:30 a.m., 10:30–11:00 a.m., and 11:00–11:30 a.m.), and 3.5 h in the afternoon (14:00–14:30 p.m., 14:30–15:00 p.m., 15:00–15:30 p.m., 15:30–16:00 p.m., 16:00–16:30 p.m., 16:30–17:00 p.m., and 17:00–17:30 p.m.). The recordings were analyzed by three trained raters blinded to the experiment design, with an inter-rater correlation coefficient of >0.90 after a period of training. The ethogram in the home cage included huddling, exploration, spontaneous locomotion, reactive locomotion, anxiety-like behaviors, stereotyped behaviors, self-grooming, and masturbating.

Huddling, measured as a depression-like behavior in monkeys, was defined as a fetal-like and self-enclosed posture with the head at or below the shoulders during the waking state (Harlow and Suomi, 1971; Shively et al., 2005; Qin et al., 2015a, b, 2019, 2016). Exploration consisted of oral or tactile manipulation of surroundings and body parts except self-grooming and masturbating (Felger et al., 2007; Davenport et al., 2008). Spontaneous locomotion included walking, running, and jumping other than stereotyped behaviors. Reactive locomotion was locomotor activities inspired by environmental stimuli. All behaviors were quantified as frequency and duration per 30-min, and the data were presented as an average of 14 days.

### Open-Field Test

The floor (2.06 × 1.80 m) of the open-field test room (2.20-m high) was equally divided into 5 × 5 rectangles with red-painted lines. The monkeys were individually transported into the test room with a transport cage. Each monkey was tested for 7 days, respectively, before and after the administration without stimulus from the experimenters (15 min/day). The test for each monkey was performed at the same time of the day to minimize the effects of time. The animals were leashed with a flexible rope to help in retreating them from the test room. The rope was long enough to reach every position in the room. Videotaped behaviors of all tests were scored by raters blinded to the treatment condition. Behaviors related to anxiety and depression were analyzed, namely, urination, defecation, spontaneous locomotion, grids crossed, exploration, motionless, stereotypical behaviors, huddling, masturbating, self-grooming, attacking, and vocalization. All behaviors were presented as the mean of seven open-field tests.

### Sampling and Measurement of Cerebrospinal Fluid Neurochemicals

The CSF samples was obtained the day before the first and the day after the last administration under ketamine (10 mg/kg) anesthesia within 10–20 min of the initial experimenter contact. Lumbar CSF was collected passively into chilled polypropylene tubes via a spinal needle inserted into the dorsolumbar vertebrae. The collected samples were immediately frozen in liquid nitrogen and stored at −80°C until assayed. The CSF samples were centrifuged at 8,000 × g for 10 min at 4°C. Concentrations of noradrenaline (NA) and its metabolite HVA, 5-hydroxyindole acetic acid (5-HIAA, the metabolite of 5-HT), as well as 3,4-dihydroxyphenylacetic acid (DOPAC, the metabolite of DA), were assessed using high-performance liquid chromatography (HPLC) (Yu et al., 2003). Samples from a given animal obtained before and after administration were paired and run in a single assay. Details about the HPLC system and procedures have been described previously (Qin et al., 2019). Data of each sample represent the average of at least two assessments.

### Statistical Analysis

All statistics and plotting were performed with GraphPad (GraphPad Prism 7.04), except that the extraction of principal components from log-transformed CSF concentrations of monoamine metabolites was conducted with SPSS (IBM SPSS Statistics 19). Two-way ANOVA with repeated measures were used to analyze the differences between the Ctrl and IFN group in sucrose preference, sucrose intake, relative sucrose intake (normalized to sucrose intake before the administration), and all measured behaviors in the home cage and the open field, with Bonferroni multiple comparisons. Pearson linear correlation was used to assess the associations among the CSF concentrations of monoamine metabolites, sucrose intake, and behaviors in the home cage and the open field. All data were presented as mean ± SD (standard deviation), and all p-values in the results were two tailed with an α level of *p* < 0.050.

## Results

### Anhedonia Induced by Long-Term Interferon-Alpha Intracerebroventricular Administration

**FIGURE 1:**
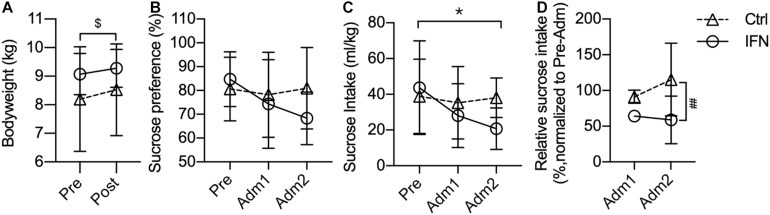
Long-term IFN-α i.c.v. administration significantly reduced the sucrose intake. The bodyweight of the Ctrl and IFN groups was comparable before and after the administration period **(A)**. Long-term IFN-α i.c.v. administration tended to decrease the sucrose preference **(B)**, and significantly reduced the sucrose intake of monkeys in the IFN group compared with the Pre phase **(C)**, as well as with the Ctrl group **(C)**. The sucrose intake was normalized to Pre for each individual animal, and they were reduced in the IFN group **(D)**. Adm1, the first 3-week of administration. Adm2, the second 3-week of administration. Ctrl, control group (*n* = 4). IFN, IFN-α administration group (*n* = 4). NS, not significant. Pre, pre-administration. Ctrl-Pre vs Ctrl-Post: ^$^*p* < 0.050. IFN-Pre vs IFN-Adm2: *p < 0.050. Ctrl-Adm2 vs IFN-Adm2: ^##^*p* < 0.010. Error bar: mean ± S D.

The bodyweight of IFN and Ctrl groups was comparable before and after the administration of IFN-α or saline (Figure 1A). The bodyweights of all animals were slightly increased after the administration, and the changes were significant in the Ctrl group (Figure 1A, ^$^*p* = 0.016).

Long-term i.c.v. IFN-α administration tended to decrease the sucrose preference (IFN group: Pre, 84.78%; Adm2, 68.34%), but the changes were not significant (Figure 1B, all *p* > 0.050). Thereafter, we compared the volume of sucrose intake, which was significantly decreased in Adm2 compared with the Pre phase in the IFN group (Figure 1C, ^∗^*p* = 0.049). In order to diminish the effect of individual difference, the sucrose intake in Adm2 was normalized to Pre for each individual animal. The sucrose intake of IFN group was reduced into 65.65 and 60.28%, respectively, in Adm1 and Adm2, and the difference between IFN group and the Ctrl group in Adm2 was significant (Figure 1D, ^#^*p* = 0.036), with a significant effect of time revealed by two-way ANOVA (*p* = 0.037). Finally, no significant change was observed in the consumption of tap water.

### Depression-Related Behaviors Induced by Long-Term Interferon-Alpha Intracerebroventricular Administration

**FIGURE 2:**
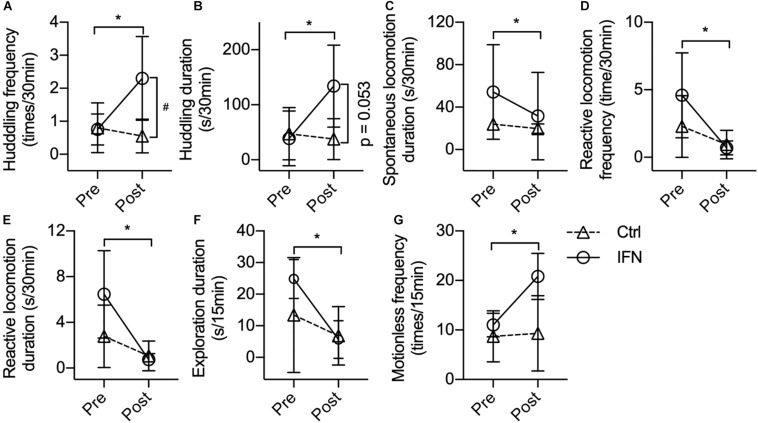
Long-term IFN-α i.c.v. administration induced depressive-like behavioral changes in rhesus monkeys in the home cage and open-field test. Six weeks of IFN-α i.c.v. administration enhanced huddling behavior. **(A,B)** while inhibiting spontaneous locomotion **(C)** and reactive locomotion **(D,E)** in the home cage. In the open-field test, IFN-α reduced the exploration duration **(F)** and increased motionless frequency **(G)** of the IFN group. Behaviors with a time scale of 30 and 15 min were observed in the home cage **(A–E)** and in the open-field **(F,G)** tests, respectively. Ctrl, control group (*n* = 4). IFN, IFN-α administration group [*n* = 4 for **(A–E)**; *n* = 3 for **(F,G)]**. Post, post-administration; Pre, pre-administration. IFN-Pre vs. IFN-Post: *p < 0.050. Ctrl-Post vs. IFN-Post: ^#^*p* < 0.050. Error bar: mean ± SD.

The IFN group showed increased post-Adm huddling frequency (Figure 2A, ^∗^*p* = 0.019) and duration (Figure 2B, ^∗^*p* = 0.037) compared with pre-Adm. The IFN group exhibited higher frequency of huddling behavior than the Ctrl group (Figure 2A, ^#^*p* = 0.021) after the IFN-α i.c.v. administration. Two-way ANOVA revealed significant effect of interaction between time and treatment on both frequency (*p* = 0.022) and duration (*p* = 0.047) of huddling behavior. Consistently, inhibited duration of spontaneous locomotion (Figure 2C, ^∗^*p* = 0.016) as well as reduced frequency (Figure 2D, ^∗^*p* = 0.044) and duration (Figure 2E, ^∗^*p* = 0.020) of reactive locomotion was observed in the IFN group after IFN-α i.c.v. administration. Finally, no significant change in exploration, anxiety-like behaviors, self-grooming, masturbating, and stereotyped behaviors was observed in either group after the i.c.v. administration of IFN-α or saline.

One monkey in the IFN group was excluded when the behaviors were analyzed in the open field because it barely moved in the test room both before and after the IFN-α administration. Different from the behaviors in home cage, no huddling behavior was observed in the open field. Long-term IFN-α i.c.v. administration significantly reduced the duration of environmental exploration of the IFN group (Figure 2F, *p* = 0.012), with a significant effect of time (*p* = 0.006) revealed by two-way ANOVA. The IFN group showed significantly higher post-Adm motionless frequency than the Ctrl group (Figure 2G, ^∗^*p* = 0.044) in the open field after 6 weeks of IFN-α administration. No significant change was observed in the frequency and duration of locomotion and grids crossed during locomotion. Finally, some behaviors including urinating, defecating, self-grooming, masturbating, attacking, or vocalization were too scarce in the open field to do a statistical analysis.

### Interferon-Alpha Intracerebroventricular Administration Increased Cerebrospinal Fluid Concentrations of Noradrenaline and 3,4-Dihydroxyphenylacetic Acid

**FIGURE 3:**
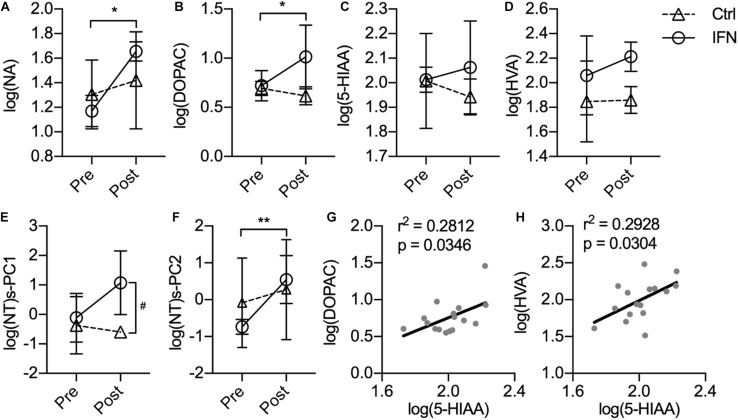
Long-term IFN-α i.c.v. administration increased the cerebrospinal fluid (CSF) concentrations of monoamine metabolites. The CSF concentrations of NA **(A)** and DOPAC **(B)** were significantly increased after IFN-α i.c.v. administration, but not 5-hydroxyindole acetic acid (5-HIAA) **(C)** and homovanillic acid (HVA) **(D)**. The principal components extracted from the log-transformed CSF concentrations of these four monoamine metabolites were also augmented by IFN-α administration **(E,F)**. The CSF concentrations of 5-HIAA showed positive correlation with those of DOPAC **(G)** and HVA **(H)**. Ctrl, control group (*n* = 4). IFN, IFN-α administration group (*n* = 4). log(NT)s-PC1 and log(NT)s-PC2, principal components derived from the log-transformed CSF concentrations of four measured monoamine metabolites. NT, neurotransmitter. Post, post-administration. Pre, pre-administration. IFN-Pre vs. IFN-Post: *p < 0.050. Ctrl-Post vs. IFN-Post: ^#^*p* < 0.050. Error bar: mean ± SD. IFN-Pre vs. IFN-Post: **p < 0.010.

The IFN group had increased the CSF concentrations of NA (Figure 3A, ^∗^*p* = 0.007) and DOPAC (Figure 3B, ^∗^*p* = 0.023) after IFN-α administration. Further way-way ANOVA revealed a significant effect of time (*p* = 0.006) and interaction between time and group (*p* = 0.044) on the CSF NA, as well as a significant effect of group (*p* = 0.022) on the DOPAC. However, the CSF concentrations of 5-HIAA (Figure 3C, *p* > 0.050) and HVA (Figure 3D, *p* > 0.050) were not significantly changed by i.c.v. administration of saline or IFN-α. In order to assess the integrated functions of monoamines on the behaviors, two principal components were derived from the log-transformed CSF concentrations of all four monoamine metabolites as log(NT)s-PC1 and log(NT)s-PC2 with principal component analysis (PCA). After long-term IFN-α i.c.v. administration, the IFN group had increased log(NT)s-PC1 compared with pre-Adm (Figure 3E, ^#^*p* = 0.031), as well as higher log(NT)s-PC2 compared with the Ctrl group (Figure 3F, ^∗∗^*p* = 0.007). Two-way ANOVA revealed significant effect of group on log(NT)s-PC1 (*p* = 0.011) and effect of time on log(NT)s-PC2 (*p* = 0.006). The CSF concentrations of 5-HIAA showed positive correlations with those of DOPAC and HVA (Figures 3G,H).

### Correlations Between the Cerebrospinal Fluid Concentrations of Monoamine Metabolites and Depression-Related Behaviors

**FIGURE 4:**
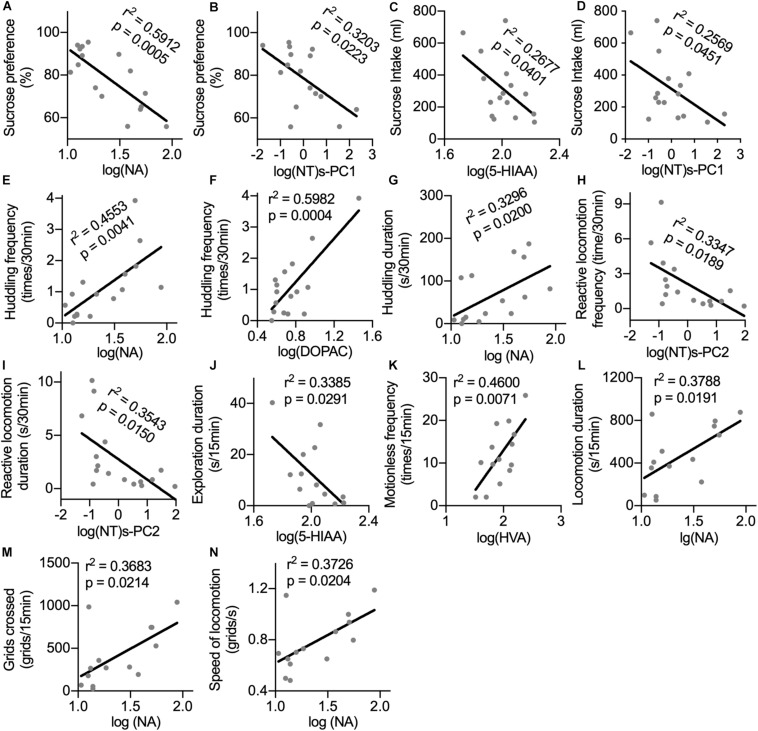
Correlations between the CSF concentrations of monoamine metabolites with anhedonia and depression-related behaviors. For the correlation analysis of the CSF monoamine metabolites with sucrose consumption **(A–D)** and behaviors in home cage **(E–I)**, the sample size was *n* = 16. For the correlation analysis between the CSF monoamine metabolites and behaviors in the open field test **(J–N)**, the sample size was *n* = 14 because one monkey was excluded from the IFN group. Behaviors with time scale of 30 and 15 min were observed in the home cage and in the open-field test, respectively. NT, neurotransmitter. log(NT)s-PC1 and log(NT)s-PC2, principal components derived from the log-transformed CSF concentrations of four measured monoamine metabolites.

Sucrose preference could be predicted by the CSF concentrations of NA and log(NT)s-PC1 with negative correlations (Figures 4A,B). Similarly, sucrose intake showed negative correlations with the CSF concentrations of 5-HIAA and log(NT)s-PC1 (Figures 4C,D, respectively). In the home cage, the CSF levels of both NA and DOPAC were strong predictors of huddling frequency (Figures 4E,F, respectively), and the duration of huddling behavior could also be predicted by the CSF NA levels (Figure 4G). Both the frequency and duration of reactive locomotion in the home cage showed a negative correlation with log(NT)s-PC2 (Figures 4H,I). In the open field, a negative correlation was found between the duration of exploration and the CSF 5-HIAA concentrations (Figure 4J). Motionless frequency in the open field was positively correlated with the CSF HVA levels (Figure 4K). Finally, the CSF concentrations of NA showed positive correlations with the locomotion duration, number of crossed grids, and speed of locomotion in the open field (Figures 4L–N).

### Correlations Between Behaviors in Different Tests

**FIGURE 5:**
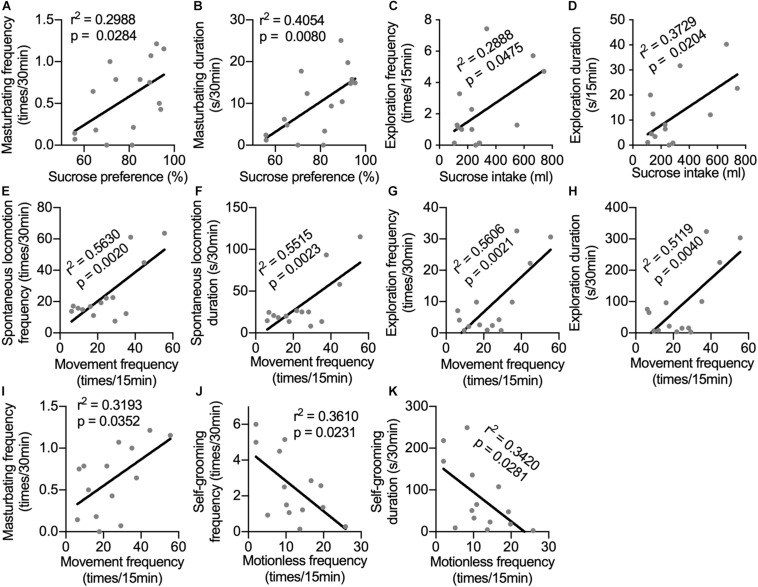
Correlations between behaviors in different tests. The correlations were analyzed between sucrose preference and frequency **(A)** and duration of masturbating behavior in home cage **(B)**; between sucrose intake and frequency **(C)** and duration of the exploration behaviors in the open field **(D)**; between movement frequency in the open field and the frequency **(E)** and duration of spontaneous locomotion **(F)**, as well as the frequency **(G)**, duration of exploration **(H)** and the masturbating frequency **(I)** in the home cage; between the frequency **(J)** and duration of the self-grooming behavior in the home cage **(K)** and the frequency of motionless in the open field. Behaviors in different tests showed significant correlations indicating that they were possibly influenced by the same mental status. Behaviors with time scale of 30-min and 15-min were observed in home cage and in the open-field test respectively.

Until now, depression-like behavioral changes induced by long-term i.c.v. administration of IFN-α have been observed in sucrose preference test, in home cage, and in the open-field test. Monkeys in the IFN group displayed different aspects of depression-related behaviors. It would be interesting to know whether behaviors displayed in different tests had some innate relation with each other. In Figure 4, we can see that some neurotransmitters showed correlations with behaviors in different tests, indicating that behaviors in different tests could be modulated by the same changes in neurotransmitters. Thus, the correlations between behaviors in different tests were analyzed. Sucrose preference was positively correlated with both frequency and duration of masturbating behavior in home cage (Figures 5A,B). Sucrose intake was positively correlated with both frequency and duration of the exploration behaviors in the open field (Figures 5C,D). The frequency of locomotion in the open field showed strong positive correlations with the frequency and duration of locomotion (Figures 5E,F) and exploration (Figures 5G,H), as well as the masturbating frequency in the home cage (Figure 5I). Finally, the frequency and duration of the self-grooming behavior in the home cage were negatively correlated with the frequency of motionless in the open field (Figures 5J,K).

## Discussion

In humans, peripheral IFN-α-induced depression is more associated with somatic symptoms and less associated with symptoms of mood and negative cognition, compared with patients with major depressive disorder (Capuron et al., 2009; Su et al., 2019). Consistently, the anxiety-like behaviors and stereotypical behaviors were not changed by IFN-α administration in this study. The increased huddling and motionless frequency, as well as decreased spontaneous locomotion and reactive locomotion were more likely to be a manifestation of depressed mood or loss of interest and motivation, rather than physical retardation (e.g., fatigue) because the mobility was not reduced in the open field. In support of this possibility, the locomotor behaviors in both home cage and the open field were positively associated with intrinsically motivated behaviors including masturbating, self-grooming, and exploratory behaviors. We also found correlations between behaviors in different tests. For example, monkeys, which showed lower sucrose preference, tended to show lower masturbating behavior in the home cage, and monkeys which showed lower sucrose intake also tended to have less exploratory behavior in the open-field test. Moreover, both sucrose intake and exploratory behavior were positively correlated with the CSF concentrations of 5-HIAA, indicating that these behaviors in the different tests could be influenced by the same changes in neurotransmitters.

Huddling behavior was observed in monkeys even before saline/IFN-α administration in this study. It was different from Felger’s study (Felger et al., 2007), possibly because of the small and isolated housing environment (Suomi and Harlow, 1972). Thus, IFN-α induced depressive-like behaviors in monkeys, but the development and expression of the depressive symptoms was also environment dependent. In support of this hypothesis, huddling behavior was only observed in the home cage, and IFN-α administration only inhibited exploration in the open field. Another interesting finding was that the association between CSF monoamine metabolites and specific behaviors only existed in one environment, in either home cage or the open field. For example, CSF concentrations of 5-HIAA was associated with the exploration duration in the open field, but not in the home cage. Then, whether and to what extent the social isolation housing environment might potentiate the development and expression of IFN-α-induced depression are interesting questions and worth further investigation. Thus, attention should be payed to the housing and evaluating/testing environment when developing non-human primate animal models of mental disorders.

In patients subcutaneously treated with IFN-α for 4–6 weeks, fMRI revealed significantly reduced bilateral activation of the ventral striatum during a reward-related task, and this change was correlated with anhedonia and depressive symptoms (Capuron et al., 2012). Anhedonia-like behavior was also observed in rhesus monkeys accompanied by decreased dopamine release and dopamine 2 receptor (D2R) binding in the striatum after 4 weeks of subcutaneous administration of IFN-α (Felger et al., 2013b). Consistently, 6 weeks of IFN-α i.c.v. administration significantly reduced the sucrose consumption, accompanied by significant change in DA metabolite DOPAC in the CSF. Besides, the change in sucrose consumption might be directly explained by the IFN-α-induced increase in CSF concentrations of 5-HIAA and NA, together with their negative correlation with sucrose consumption and sucrose preference.

Although IFN-α i.c.v. administration only significantly changed the CSF concentrations of NA and DOPAC, the correlations between all measured monoamine metabolites and depression-related behaviors indicated their involvement in regulating these behaviors. Moreover, the correlations of the principal components derived from the CSF concentrations of these monoamine metabolites with depression-related behaviors suggested that some behaviors could possibly be mediated by multiple neurotransmitters.

The concentrations of neurotransmitters and their metabolites in the CSF were dependent on the balance between synthesis and metabolism. Although the 5-HIAA concentrations in the CFS was not significantly changed by IFN-α i.c.v. administration in this study, a change in functional 5-HT levels in the brain was still possible. IFN-α induces depression by activating IL-6 (Schaefer et al., 2003), which disrupts the function of tetrahydrobiopterin (BH4) in the synthesis of 5-HT (Felger and Lotrich, 2013). IL-6 can also inhibit the 5-HT synthesis via induction of indoleamine-2,3-dioxygenase (IDO) enzyme, which facilitates the metabolism of tryptophan into kynurenine (KYN) (Anderson et al., 2013). For example, a single i.c.v. IFN-α injection in rats significantly reduced the 5-HT levels in the frontal cortex, midbrain, and stratum of rats in a dose-dependent manner (Kamata et al., 2000).

It was found in mice that repeated peripheral administration of IFN-α induced depressive-like behaviors via downregulating the serotoninergic receptor 5-HT1A and promoting apoptosis in the hippocampus (Ping et al., 2012). Increase in IFN-α and serotonin transporter (SERT) expression happen together in central inflammation (Katafuchi et al., 2005), and IFN-α administration itself has been found to stimulate SERT expression (Schaefer et al., 2003). In both situations, the 5-HT uptake from the synapses is enhanced (Tsao et al., 2008). However, with upregulated 5-HT reuptake, the CSF concentrations of 5-HIAA should be decreased instead of unchanged as observed in the current study. A more plausible possibility was that IFN-α increased the metabolism of 5-HT into 5-HIAA (Schaefer et al., 2003), leading to an increased 5-HIAA/5-HT ratio (De La GarzaII, and Asnis, 2003) via inhibiting SERT function (Foley et al., 2007) and increasing platelet monoamine oxidase-B (MAO) activities (Bannink et al., 2005). The result will induce reduced recycling of 5-HT, even less material for 5-HT synthesis, and finally a deficiency of functional 5-HT in the synapses. In support of this possibility, decreased sucrose preference and exploratory behaviors, as well as increased immobility in forced swim test was observed in SERT knock-out mice and rats (Olivier et al., 2008; Popa et al., 2008). Patients with long-form of the SERT gene (L_A_/L_A_ genotype) are more resistant to IFN-α-induced depression (Lotrich et al., 2009).

Similarly, peripheral IFN-α has been found to decrease the DA functions in the brain by inhibiting the conversion of the phenylalanine (Phen) to Tyr, the primary amino acid precursor of DA (Felger and Miller, 2012; Felger et al., 2013a). Moreover, rhesus monkeys receiving 4 weeks of IFN-α subcutaneous injections exhibited decreased striatal DA release and DA binding to receptors D2R, without change in the DA transporter (Felger et al., 2013b). However, significantly increased DA uptake and decreased DA turnover in caudate, putamen, and ventral striatum was observed in human patients receiving IFN-α for 4–6 weeks (Capuron et al., 2012). These changes in DA functions were similar to the 5-HT changes in peripheral IFN-α administration and might contribute to the IFN-α-induced anhedonia and depression. Nevertheless, the CSF concentrations of DOPAC were upregulated by i.c.v. IFN-α administration in this study, possibly because of increased metabolism of DA.

In conclusion, we hypothesize that i.c.v. IFN-α administration reduced the synthesis and increased the metabolism of 5-HT and DA, leading to a deficiency in functional 5-HT and DA in the brain (Kamata et al., 2000). It can explain the positive correlations of CSF concentrations of 5-HIAA and DOPAC with the severity of depression in rhesus monkeys in this study. The correlations of the CSF concentrations of 5-HIAA with DOPAC and HVA was probably a result of dose-dependent activation of monoamine metabolism by IFN-α. Nevertheless, the functioning and metabolism of NA is relatively special and more complicated among these monoamines because NA can be taken up by the DAT of dopaminergic neurons (Haenisch and Bönisch, 2011). It means that the depletion of noradrenaline transporters (NATs) can be partially compensated by other monoamine transporters, retaining the proportion between NA in the CSF and functional NA in the brain. The recruitment of DATs by NA further decreased the recycling/turnover of DA, making NA positively correlated with the severity of depressive symptoms including anhedonia and huddling behavior. Another possibility was that NA directly promoted depressive symptoms. In genetically modified mice, increased NA levels in the brain was accompanied with decreased sucrose preference and intake (Aizawa et al., 2016).

The mechanisms about how peripheral IFN-α regulates the metabolism and functioning of monoamines are relatively well studied (Felger and Miller, 2012). However, the behavioral and physical effects of both long-term and acute IFN-α i.c.v. administration have been scarcely studied. Little is known about how IFN-α in the CNS changes the process of monoamine synthesis, packaging, release, reuptake and metabolism, let alone how these changes contribute to the development of IFN-induced depression.

Accumulating studies have found that some possible mechanisms were involved in the development of IFN-α-induced depression, including dysregulation of monoaminergic and glutamatergic neurotransmitter systems (Capuron et al., 2003; Haroon et al., 2014), alterations of activities of the HPA axis (Felger et al., 2016), and impairment of neuronal survival and plasticity (Hoyo-Becerra et al., 2014; Zheng et al., 2015). However, most of these observations was obtained from patients with preexisting health problems or laboratory animals receiving IFN-α treatment through peripheral administration. However, the central nervous system is separated from the circulating blood by the blood–brain barrier. In rhesus monkeys, peripherally administrated IFN-α can hardly be detected in the CSF; on the other hand, the IFN-α in plasma is not detectable following an i.c.v. administration (Collins et al., 1985). Specifically, the initial CSF concentrations of IFN-α exceeded 100,000 U/ml with a dose of 120,000 U/kg i.c.v. administration and remained at levels above 100 U/ml for 2 days. Central administration of IFN-α provided a total exposure (area under the concentrations vs. time curve) for the CSF 3,000-fold greater than peripheral administration with a 20-fold larger dose (Collins et al., 1985). Thus, studies with central IFN-α administration are urgently necessary to answer these pivotal questions.

This animal model is the first non-human primate model of depression induced by central administration of IFN-α, which is necessary to explore the mechanisms of depression induced by i.c.v. IFN-α treatment and the CNS inflammation. Rhesus monkeys showed considerable depressive-like symptoms accompanied with changes in the CSF concentrations of monoamine metabolites after 6 weeks of i.c.v. IFN-α administration. Anhedonia was observed as significantly reduced sucrose intake during the IFN-α administration. Monkeys receiving i.c.v. IFN-α infusion spent more time huddling in a corner of their home cages and showed less interests in their physical and living environment even when they were stimulated. Bodyweights of both Ctrl and IFN groups were increased, suggesting that the i.c.v. administration of IFN-α or saline with the dose in this study did not cause health problem to these monkeys. In other words, the behavioral changes in the IFN group was not a result of IFN-α-induced sickness. This study also found that increased metabolism of monoamines in the brain might play an important role in central IFN-α-induced depression. Thus, even though both peripheral and central administration of IFN-α can induce depressive symptoms in humans and animal models, the underlying mechanisms and involved pathways are likely to be different. Restoration of normal monoamine metabolisms in the brain is a potential therapeutic strategy to prevent and cure depression induced by central IFN-α and CNS inflammation.

## Data Availability Statement

All datasets generated for this study are included in the article/supplementary material, further inquiries can be directed to the corresponding authors.

## Ethics Statement

The animal study was reviewed and approved by the National Animal Research Authority (China) and the Institutional Animal Care and Use Committee (IACUC) of Kunming Institute of Zoology, Chinese Academy of Sciences.

## Author Contributions

All authors listed have made a substantial, direct and intellectual contribution to the work, and approved it for publication.

## Conflict of Interest

The authors declare that the research was conducted in the absence of any commercial or financial relationships that could be construed as a potential conflict of interest.
